# MFG-E8 Plays an Important Role in Attenuating Cerulein-Induced Acute Pancreatitis in Mice

**DOI:** 10.3390/cells10040728

**Published:** 2021-03-25

**Authors:** Heng-Fu Bu, Saravanan Subramanian, Hua Geng, Xiao Wang, Fangyi Liu, Pauline M. Chou, Chao Du, Isabelle G. De Plaen, Xiao-Di Tan

**Affiliations:** 1 Division of Pediatric Gastroenterology, Hepatology, and Nutrition, Department of Pediatrics, Center for Intestinal and Liver Inflammation Research, Ann & Robert H. Lurie Children’s Hospital of Chicago, Chicago, IL 60611, USA; hbu@luriechildrens.org (H.-F.B.); ssubramanian@luriechildrens.org (S.S.); hgeng@luriechildrens.org (H.G.); xiwang@luriechildrens.org (X.W.); fangyi_nj@126.com (F.L.); chao.du@northwestern.edu (C.D.); 2 Department of Pediatrics, Feinberg School of Medicine, Northwestern University, Chicago, IL 60611, USA; ideplaen@luriechildrens.org; 3 Department of Pathology, Feinberg School of Medicine, Northwestern University, Chicago, IL 60611, USA; pchou@luriechildrens.org; 4 Division of Neonatology, Department of Pediatrics, Ann & Robert H. Lurie Children’s Hospital of Chicago, Chicago, IL 60611, USA; 5 Department of Research & Development, Jesse Brown Veterans Affairs Medical Center, Chicago, IL 60612, USA

**Keywords:** MFG-E8, cerulein-induced acute pancreatitis, *Mfge8* knockout mouse

## Abstract

Milk fat globule-EGF factor 8 (MFG-E8) is a secreted glycoprotein that regulates tissue homeostasis, possesses potent anti-inflammatory properties, and protects against tissue injury. The human pancreas expresses MFG-E8; however, the role of MFG-E8 in the pancreas remains unclear. We examined the expression of MFG-E8 in the pancreas at baseline and during cerulein-induced acute pancreatitis in mice and determined whether MFG-E8 attenuates the progression of pancreatitis, a serious inflammatory condition that can be life-threatening. We administered cerulein to wild-type (WT) and *Mfge8* knockout (KO) mice to induce pancreatitis. Immunoblot analysis showed that MFG-E8 is constitutively expressed in the murine pancreas and is increased in mice with cerulein-induced acute pancreatitis. In situ hybridization revealed that ductal epithelial cells in the mouse pancreas express *Mfge8* transcripts at baseline. During pancreatitis, *Mfge8* transcripts were abundantly expressed in acinar cells and endothelial cells in addition to ductal epithelial cells. Knocking out *Mfge8* in mice exacerbated the severity of cerulein-induced acute pancreatitis and delayed its resolution. In contrast, administration of recombinant MFG-E8 attenuated cerulein-induced acute pancreatitis and promoted repair of pancreatic injury in *Mfge8* KO mice. Taken together, our study suggests that MFG-E8 protects the pancreas against inflammatory injury and promotes pancreatic tissue repair. MFG-E8 may represent a novel therapeutic target in acute pancreatitis.

## 1. Introduction

Acute pancreatitis is an inflammatory process of the pancreas [1,2]. Clinical manifestations of acute pancreatitis vary from mild to moderately severe to severe and life-threatening. Overall mortality ranges from 2% to 20%, depending on severity [1,2]. Decades of research have advanced our understanding of the pathogenesis and pathophysiological mechanisms underlying acute pancreatitis [3]; however, there are no targeted pharmacological therapies for acute pancreatitis and specific therapeutic approaches remain limited [3,4]. To date, supportive care is the primary treatment for acute pancreatitis [5,6].

During the past ten years, investigators have revealed several novel potential therapeutic targets in experimental models of pancreatitis. For example, the neuronal guidance protein, netrin-1, has been shown to have anti-inflammatory effects in mice with severe acute pancreatitis [7]. Recent studies demonstrated that galactose and channel inactivator store-operated calcium entry-associated regulatory factor (SARAF) play a role in preventing pancreatic damage during development of acute pancreatitis [8,9]. In addition, pancreatic signal transducer and activator of transcription 3 (STAT3) has been found to protect mice against cerulein-induced pancreatitis [10]. Furthermore, Sakuma et al. showed that the chemokine CXCL16 mediates acinar cell necrosis in cerulein-induced acute pancreatitis in mice [11]. Together, these advances have shed new light on the potential for targeted prevention and treatment of acute pancreatitis.

Milk fat globule-EGF factor 8 (MFG-E8), also known as lactadherin in humans, is an anti-inflammatory glycoprotein produced in various types of cells in vivo [12,13]. Initially, MFG-E8 was found to bind to phosphatidylserine on apoptotic cells and interact with αvβ3 integrin on macrophages to enhance engulfment of apoptotic cells [14]. In addition to facilitating apoptotic cell clearance, we and others previously showed that MFG-E8 possesses a wide range of other physiological activities such as promoting intestinal epithelial restitution and enhancing angiogenesis [15,16,17,18]. MFG-E8 has been found to play an important role in protecting against sepsis, attenuating inflammatory responses and tissue injury, and promoting wound healing and tissue regeneration [15,16,17,19,20,21,22]. Here, we hypothesize that MFG-E8 also protects against acute pancreatitis.

To test this hypothesis, we examined the role of MFG-E8 in pancreatic inflammation using an animal model of pancreatitis and *Mfge8*-deficient mice. We found that MFG-E8 protein expression is induced by acute pancreatitis, loss of MFG-E8 expression is associated with disease progression and delayed recovery, and treatment with MFG-E8 can attenuate pancreatic injury. Our data suggest that MFG-E8 is a potential therapeutic target for the treatment of patients with acute pancreatitis.

## 2. Materials and Methods

### 2.1. Animals

C57BL/6J wild-type (WT) mice were purchased from Jackson Laboratory (Bar Harbor, ME). *Mfge8* knockout (KO) mice (fully congenic with the C57BL/6 background) were obtained as previously described [15,16]. Animals were housed in a specific pathogen-free animal facility at our institution. Water and standard chow (Cat# 7912, Envigo, Denver, CO, USA) were provided ad libitum throughout the study. Male mice (8–10 weeks old) were used in this study. All animal experiments were conducted per NIH guidelines and protocols approved by the Institutional Animal Care and Use Committee of our institution.

### 2.2. Cerulein-Induced Acute Pancreatitis Model

Cerulein (Sigma-Aldrich, St. Louis, MO) was dissolved in sterile saline. Acute pancreatitis was induced in unstarved mice by giving 10 hourly intraperitoneal (IP) injections of 50 μg/kg body weight cerulein [23]. Sham-controls were injected IP with an equal volume of sterile saline. To test the role of MFG-E8 in the pathogenesis of pancreatitis, recombinant mouse MFG-E8 (rMFG-E8, 20 μg/kg body weight; R&D Systems, Minneapolis, MN) was injected IP to *Mfge8* KO mice 2 h before the first cerulein injection, then twice a day for 7 days to rescue MFG-E8 deficiency. This MFG-E8 dose was selected based on our previous observations [16]. At 11 h, 24 h, 2 days, 4 days and 7 days after the first cerulein injection, mice were euthanized using CO_2_ inhalation followed by bilateral pneumothorax or cervical dislocation, and the pancreas and blood samples were collected. The pancreas was either snap-frozen in liquid nitrogen and stored at –80 °C for molecular studies or fixed in 10% formalin and paraffin-embedded for histopathological examination. The plasma was separated from blood by centrifugation and stored at –80 °C for analysis of circulating MFG-E8 levels with ELISA.

### 2.3. Histological Examination

After fixation in 10% buffered formalin for 24 h, pancreatic tissues were processed for routine histology. Tissue sections (5 μm) were stained with hematoxylin and eosin (H&E). Histological examination was performed by a blinded investigator using scoring systems modified from previously described methods [23,24,25,26]. Murine cerulein-induced acute pancreatitis is composed of an acute phase (between 11–24 h after the first cerulein injection) and a recovery phase (from Day 2 to Day 7 after the first cerulein injection) [27]. To determine the severity of the acute phase, H&E-stained pancreatic tissue sections were graded using the criteria in Table 1.

To determine the efficiency of pancreatic tissue repair (recovery phase), sections were examined and the percentage of regenerated acini in injured pancreatic lobules was estimated using a scoring system described in Table 2.

### 2.4. Immunoblot Analysis

Total protein was isolated from mouse pancreas using a standard protocol described previously [15,28]. Protein (100 μg/sample) was loaded and electrophoresed in sodium dodecyl sulfate polyacrylamide gel electrophoresis gels, then transferred onto polyvinylidene difluoride membranes. Immunoblot analysis was carried out using antigen affinity-purified polyclonal goat IgG against murine MFG-E8 (1:800; AF2805, R&D Systems) as a primary antibody and horseradish peroxide-conjugated donkey anti-goat IgG (1:3000) as a secondary antibody. After additional washing with phosphate-buffered saline containing 0.1% Tween 20, immune complexes were visualized using Pierce™ ECL Western Blotting Substrate (Thermo Fisher Scientific, Waltham, MA, USA). β-Actin protein was used as the loading control. Band intensity was measured by densitometric analysis using ImageJ, a Java-based image-processing program available by the National Institutes of Health.

### 2.5. RNAscope Fluorescent In Situ Hybridization Assay

Formalin-fixed and paraffin-embedded tissue sections (5-µm thickness) were used with the RNAscope Fluorescent Reagent Kit v2 and an RNAscope probe for mouse *Mfge8* mRNA (Cat# 408771, Advanced Cell Diagnostics, Newark, CA, USA). A standard protocol used by our laboratory [29] for RNAscope fluorescent in situ hybridization was followed. After staining, slides were mounted with the antifade mounting media containing 4′, 6-diamidino-2-phenylindole (DAPI, H-1500, Vector Laboratories, Burlingame, CA, USA) and images were acquired using THUNDER Imaging Systems (Leica Microsystems, Wetzlar, Germany). Images were processed with Adobe Photoshop software (CC 2020).

### 2.6. ELISA

MFG-E8 concentration in plasma samples was measured using a mouse MFG-E8 ELISA immunoassay kit (MFGE80, R&D Systems), following the manufacturer’s protocol. The detection range of the assay is 0.125–8.0 ng/mL. All the samples were measured in duplicate.

### 2.7. Statistical Analysis

Statistical analysis was performed with GraphPad Prism 6 software. An independent Student’s t-test and ANOVA analysis of one-way variance followed by multiple comparisons test were used to assess the significance of group differences. *p* < 0.05 was considered statistically significant. Data are shown as mean ± standard error of the mean (SEM).

## 3. Results

### 3.1. MFG-E8 Expression Is Increased in the Exocrine Cellular Compartment of Mouse Pancreas during Cerulein-Induced Acute Pancreatitis

**Figure 1:**
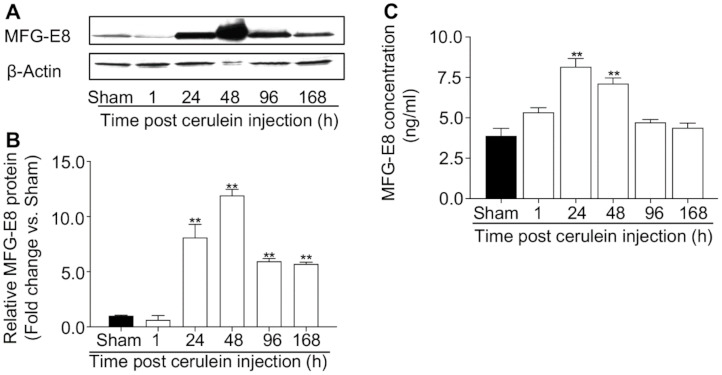
Cerulein-induced acute pancreatitis is associated with upregulation of MFG-E8. (**A**,**B**) Young adult C57BL/6J mice (male, 8–10 weeks old) were subjected to 10 hourly intraperitoneal (IP) injections of cerulein (50 μg/kg) and euthanized at the timepoints indicated. Mice in the sham-group were treated with saline. Pancreatic tissue was collected and processed for MFG-E8 immunoblot analysis. Panel A shows a representative immunoblot and panel B shows densitometric analysis of the immunoblot data (normalized to β-Actin, *n* = 3 per time point). ** *p* < 0.01 versus sham. (**C**) Plasma samples were collected at the indicated timepoints and processed to quantify MFG-E8 levels by ELISA. *n* = 6 each time point. ** *p* < 0.01 versus sham controls.

Repeated administration of cerulein induces self-limited acute pancreatitis in mice [23,24,25,26,27], with a natural course divided into an acute phase (up to 24 h after the first injection) and a recovery phase (2 to 7 days after the first injection) [27]. Utilizing this model, we examined MFG-E8 expression during both the acute and recovery phases by immunoblot. MFG-E8 was constitutively expressed in mouse pancreas. Its expression increased in the late acute phase (24 h), reached its peak 48 h after cerulein challenge, and declined during the recovery phase but remained at a higher level compared to sham-controls (Figure 1A,B).

MFG-E8 is a secreted protein [30]; therefore, we also measured MFG-E8 concentration in the plasma by ELISA. Similar to the changes seen in pancreatic tissue, plasma levels of MFG-E8 were markedly increased at 24 h after cerulein administration (Figure 1C), declined at 48 h, and returned to baseline thereafter.

**Figure 2:**
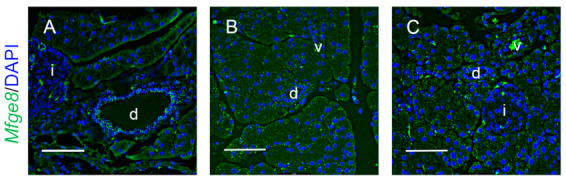
Cellular localization of *Mfge8* transcripts in the pancreas of mice with acute pancreatitis using RNAscope fluorescent in situ hybridization assay. Young adult C57BL/6J mice (male, 8–10 weeks old) were subjected to cerulein treatment for induction of acute pancreatitis as described in Figure 1. Pancreatic tissue was fixed with 10% formalin and processed for routine histology. Sections of pancreas were stained using the RNAscope^®^ Fluorescent Assay with a probe for *Mfge8* transcripts (green) followed by counterstaining with DAPI (blue). Representative fluorescent microscopy images show the localization of *Mfge8* transcripts in pancreas of mice in the sham-treated group (**A**), 24 h post-cerulein injection (**B**), and 48 h post-cerulein injection (**C**); d indicates ducts; v, blood vessels; and i, islets in the pancreatic lobules. Scale bar: 50 μm.

Next, we examined which cells produce MFG-E8 in mouse pancreas during acute pancreatitis using RNAscope fluorescent in situ hybridization assay. *Mfge8* was constitutively expressed in ductal epithelial cells in the pancreas of sham-treated mice (Figure 2A). At 24 h after induction of pancreatitis by cerulein, *Mfge8* mRNA signals were localized to ductal epithelial cells and acinar cells in pancreas (Figure 2B). At 48 h, *Mfge8* gene expression was induced not only in acinar cells and ductal epithelial cells but also in endothelial cells of interlobular blood vessels (Figure 2C), suggesting that MFG-E8 is diffusely present in pancreatic tissue during the late acute and recovery phase of acute pancreatitis. However, in situ hybridization did not reveal *Mfge8* gene expression in pancreatic islets of sham-treated mice or during cerulein-induced pancreatitis (Figure 2).

Taken together, these data suggest that cerulein-induced pancreatitis is associated with induction of MFG-E8 in various cells in the exocrine pancreas after the onset of inflammation and throughout the recovery phase.

### 3.2. MFG-E8 Deficiency Exacerbates Cerulein-Induced Acute Pancreatitis in Mice

We next investigated the role of MFG-E8 in the pathogenesis of cerulein-induced acute pancreatitis using *Mfge8* KO mice. Histologic examination of the pancreas in *Mfge8* KO mice did not reveal any phenotypical abnormalities (Supplementary Figure S1), suggesting that MFG-E8 is not an essential protein for the development and the homeostasis of pancreatic tissues under physiological conditions.

**Figure 3:**
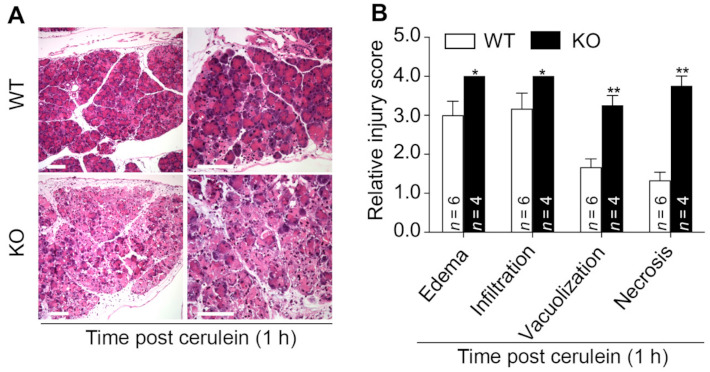
*Mfge8* knockout (KO) mice develop more severe cerulein-induced acute pancreatitis than wild type (WT) mice. WT and *Mfge8* KO mice (male, 8–10 weeks old) were administered 10 hourly doses of cerulein to induce acute pancreatitis. Animals were euthanized 1 h after the last cerulein treatment. (**A**) Representative images of H&E-stained pancreatic tissue slides. (**B**) Histopathology scores of indicated inflammatory parameters were quantified as described in Table 1. * *p* < 0.05 and ** *p* < 0.01 versus cerulein-treated WT mice. Scale bar: 100 μm.

To understand the role of MFG-E8 in the development of pancreatitis, we compared the severity of cerulein-induced acute pancreatitis in *Mfge8* KO vs. WT mice administered 10 hourly IP injections of cerulein (50 μg/kg). Mice were euthanized 11 h after the first cerulein injection (i.e., 1 h after the last cerulein treatment). WT mice developed self-limited acute pancreatitis characterized by histological evidence of vacuolization of acinar cells, interlobular and intralobular edema, focal acinar necrosis, and perivascular and scarce diffuse infiltration of inflammatory cells (Figure 3A,B). In contrast, *Mfge8* KO mice developed histological evidence of diffuse acinar necrosis as well as more profound edema, vacuolization, and inflammatory cell infiltration in the pancreas (Figure 3A,B).

### 3.3. Mice Lacking MFG-E8 Show Delayed Recovery from Cerulein-Induced Acute Pancreatitis

**Figure 4:**
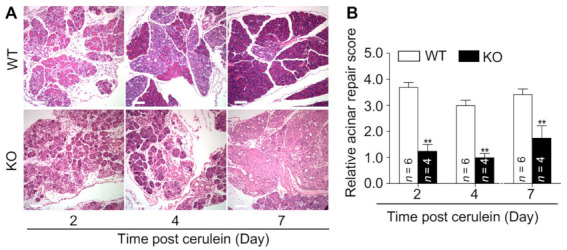
*Mfge8* KO mice exhibit delayed recovery of pancreatic injury in cerulein-induced acute pancreatitis. WT and *Mfge8* KO mice (male, 8–10 weeks old) were subjected to cerulein treatment as described in Figure 1. Animals were euthanized at indicated timepoints. (**A**) Representative histological images of pancreatic tissues (10×, H&E stain). (**B**) Acinar cell repair was quantified in formalin-fixed pancreatic sections stained with H&E based on the scoring system in Table 2. ** *p* < 0.01 versus cerulein-treated WT mice. Scale bar: 100 μm.

Previous studies showed that cerulein-induced acute pancreatitis is transient and self-limited in mice [31,32]. Because MFG-E8 levels remained high during the recovery phase of cerulein-induced acute pancreatitis (Figure 1), we examined whether lack of MFG-E8 impacts pancreatic recovery. WT and *Mfge8* KO mice were injected with cerulein as described above and the pancreas was collected at 2, 4, and 7 days after the first injection. Two days after the onset of pancreatitis, the WT mouse pancreas exhibited typical features of prominent histological stromal reaction. Profound acinar regeneration was observed at 4 days and restoration of pancreatic architecture was evident at 7 days (Figure 4A,B, and Supplementary Figure S2). In *Mfge8* KO mice, the stromal response began at 2 days and was prolonged for 7 days after the initial cerulein injection (Figure 4A,B and Supplementary Figure S2). Only mild acinar regeneration was observed throughout the course of cerulein-induced pancreatitis in *Mfge8* KO mice. Collectively, these findings demonstrate that MFG-E8 plays an important role in promoting acinar repair.

### 3.4. Recombinant MFG-E8 Compensation Decreases the Severity of Cerulein-Induced Acute Pancreatitis and Confers Acinar Repair of Pancreatitis-Associated Pancreatic Injury in Mfge8 KO Mice

**Figure 5:**
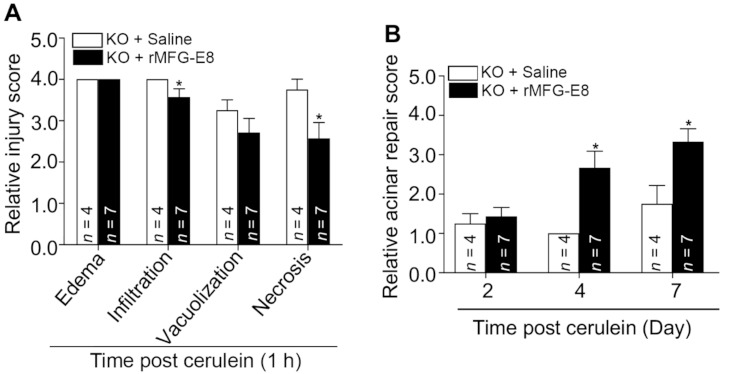
Recombinant mouse MFG-E8 protein (rMFG-E8) attenuates tissue injury and promotes acinar repair in *Mfge8* KO mice with cerulein-induced acute pancreatitis. *Mfge8* KO mice (male, 8–10 weeks old) were treated with saline or rMFG-E8 (20 μg/kg, IP) 2 h before the first cerulein injection, then twice a day for 7 days. Animals were euthanized at 1 h after the last cerulein treatment (**A**) or at the indicated timepoints (**B**). The pancreas was processed for histological examination and the relative injury score (A) and acinar repair score (B) are presented here, based on the scoring systems in Table 1 and Table 2, respectively. * *p* < 0.05 versus the cerulein-treated *Mfge8* KO + saline group.

Next, we examined whether the administration of recombinant MFG-E8 (rMFG-E8) can rescue the delayed recovery phenotype of *Mfge8* KO with cerulein-induced acute pancreatitis. *Mfge8* KO mice were injected with rMFG-E8 or saline (control) and then subjected to experimental acute pancreatitis with cerulein injections. Animals were euthanized at 11 h and 2, 4, and 7 days after the first cerulein injection. *Mfge8* KO mice treated with cerulein and saline developed severe acute pancreatitis characterized by diffuse severe edema, marked inflammatory cell infiltration, and abundant vacuolization and necrosis in 11 h after the first cerulein injection. In contrast, *Mfge8* KO mice treated with rMFG-E8 exhibited less severe inflammatory cell infiltration and reduction of acinar cell necrosis in the pancreas (Figure 5A), suggesting that rMFG-E8 attenuated cerulein-induced acute pancreatitis in *Mfge8* KO mice. Furthermore, during the recovery phase, treatment of cerulein-challenged *Mfge8* KO mice with rMFG-E8 promoted removal of necrotic debris, establishment of parenchyma structure, and diminishment of inflammation. Cerulein-challenged *Mfge8-*deficient mice treated with saline exhibited impairment of pancreatic regeneration (Figure 5B). Taken together, these data indicate that MFG-E8 plays a critical role in preventing inflammation and promoting acinar regeneration in the pancreas.

## 4. Discussion

MFG-E8is a secreted glycoprotein that orchestrates diverse biological functions [12,13]. MFG-E8 is expressed in the human pancreas [33]; however, its function in the pancreas remains unknown. Acute pancreatitis is an inflammatory disease of the pancreas that also involves peripancreatic tissues in serious cases, causing sudden severe abdominal pain [1,2]. Emerging evidence shows that the spectrum of acute pancreatitis ranges from mild to severe, and it can be fatal [1,2]. Although the majority of cases are mild and exhibit self-limiting course of illness, about 20% of patients with acute pancreatitis will develop a severe form that is often associated with life-threatening complications, such as multiple organ failure and infection [1,2]. In the present study, we found that MFG-E8 is constitutively expressed in ductal epithelial cells in the murine pancreas. Its expression is maintained in ductal epithelial cells and activated in pancreatic acinar cells and endothelial cells during development of cerulein-induced acute pancreatitis. Furthermore, we found that mice with MFG-E8 deficiency experienced more severe pancreatitis after cerulein challenge compared to WT mice. Administration of rMFG-E8 rescued *Mfge8* KO mice from cerulein-induced severe acute pancreatitis. Together, our data suggest that up-regulation of MFG-E8 plays an important role in attenuating the progression from mild to severe acute pancreatitis.

Numerous cell types express MFG-E8 in vivo. Nakatani et al. previously reported that MFG-E8 is abundantly expressed in the mammary gland epithelium [34], and we and others found that monocytes/macrophages in the mouse intestinal lamina propria express MFG-E8 [15,21]. MFG-E8 protein was found in the epithelium of the human endometrium [35], and Ensslin and Shur revealed that mouse spermatogenic cells express MFG-E8 in vivo [36]. A previous immunohistochemical analysis showed MFG-E8 could be detected in endothelial and smooth muscle cells of a number of capillaries and arterioles in mice [18]. In the present study, we found that ductal epithelial cells in mouse pancreas express *Mfge8* gene at baseline; however, MFG-E8 does not appear to be required for growth or maintenance of pancreatic tissue homeostasis under physiological conditions, as MFG-E8-deficient mice develop normally [36] and possess normal pancreatic tissue structure (Supplementary Figure S1).

Evidence suggests that MFG-E8 is an inflammation-associated protein [15,19,37] that plays an important role in maintaining tissue homeostasis during inflammation. In our previous studies, we found that MFG-E8 promotes intestinal epithelial homeostasis, enterocyte migration, and mucosal healing [15,16]. Similarly, several reports demonstrated that MFG-E8 attenuates colitis [16,21]. In addition to its protective role in intestinal tissues, MFG-E8 has been shown to regulate osteoclast homeostasis and protect against inflammatory bone loss [38]. Treatment with MFG-E8 reduces inflammation and lung injury [39], and MFG-E8/lactadherin was found to enhance neovascularization and promote wound healing [17,18]. In the present study, we found that *Mfge8* expression is not only sustained in ductal epithelial cells but activated in acinar cells as well as endothelial cells of interlobular blood vessels during acute pancreatitis. However, we did not note any change in *Mfge8* gene expression in the pancreatic islets in mice with acute pancreatitis. To our knowledge, this is the first study to report the cellular localization of MFG-E8 in the mouse pancreas. Acinar and ductal cells in the exocrine pancreas form a close functional unit. Our study demonstrated that these cells produce MFG-E8, which in turn promotes pancreatic wound healing and resolution of pancreatitis. Our novel finding advances our understanding of how these cells contribute to repair of the pancreas from inflammatory injury.

The exocrine pancreas possesses an intrinsic capacity for regeneration, which can make a rapid and full recovery from acute pancreatitis [40]. Previous studies have demonstrated that several transcription factors and pathways are required for the exocrine regenerative response to pancreatitis. Fendrich et al. reported that Hedgehog signaling plays an essential role in effective regeneration of the exocrine pancreas after acute pancreatitis [41], and Siveke et al. found that the interaction between Notch and Wnt signaling mediates the regeneration and maturation process of acinar cells [42]. In addition, transcription factor PTF1A55 has been shown to maintain the identity of pancreatic acini [43]. These studies suggest that multiple factors contribute to maintaining and reestablishing the homeostasis of the exocrine pancreas after acute pancreatitis. In the present study, we found that acinar regeneration after pancreatitis is impaired in *Mfge8* KO mice, and that treatment of these mice with rMFG-E8 partially protects against pancreatic injury during the acute phase and restores acinar regeneration and repair. Our results suggest that MFG-E8 is a critical factor that contributes to the recovery of the pancreas from inflammatory injury, though the underlying mechanisms are unknown. Previous studies showed that MFG-E8 promotes clearance of apoptotic cells by bridging phosphatidylserine on apoptotic cells and integrin αvβ3/5 on phagocytes [14]. It has also been shown to enhance restoration of homeostasis in epithelial tissues and promote angiogenesis [15,16,17,18]. Furthermore, Aziz et al. found that MFG-E8 attenuates neutrophil infiltration in inflammation via modulation of CXCR2 [37]. Several investigators previously revealed the link between STAT3 signaling and MFG-E8 anti-inflammatory function [44,45]. Recently, it has been reported that MFG-E8 plays a key role in macrophage reprogramming in tissue healing [46]. Thus, it is likely that MFG-E8 promotes the recovery of pancreas from inflammatory injury through complex mechanisms that remain to be examined in the future.

Our study has two limitations. First, although we found that MFG-E8 plays a role in blocking progression of cerulein-induced acute pancreatitis from mild to severe stages, the pharmacological and pharmacokinetic characteristics (such as the dose response, bioavailability, distribution, and metabolism) of MFG-E8 remains unknown. Second, we used a single model of cerulein-induced pancreatitis in this study. We chose this model because it is the most widely used experimental animal model of acute pancreatitis [31]. This model allows investigation of healing and regeneration of damaged acinar tissue after pancreatitis [31]. During preparation of the 2nd revision of this paper, Ren et al. published a report which suggested that MFG-E8 is also protective in an L-arginine-induced pancreatic injury model [47]. Their results are consistent with ours, and the similar finding in two different models of pancreatitis further enhances confidence in our conclusion that MFG-E8 plays an important role in attenuating acute pancreatitis. On the other hand, neither cerulein model nor L-arginine model of rodent pancreatitis can mimic the etiology of the disease in humans. It will be necessary to verify the role of MFG-E8 in other animal models of experimental pancreatitis prior to initiating preclinical and pharmacological studies of MFG-E8‒based therapy.

In summary, we report for the first time that MFG-E8 is induced during acute pancreatitis and plays a critical role in protecting against progression of acute pancreatitis and in promoting recovery of damaged pancreatic tissue. MFG-E8 may be a promising therapeutic target to attenuate the progression of pancreatitis to severe stages and enhance the repair of pancreatic tissues following inflammatory injury.

## Figures and Tables

**Table 1:** Histological scoring system for acute injury phase in mice with cerulein-induced acute pancreatitis (11–24 h after initial injection).

Score	Description of Criteria (20× Objective Field)
	**Severity of inflammation: edema**
0	Absent
1	Minimal: focally increased among lobules
2	Mild: <25% diffused expansion of among lobules
3	Moderate: 25–50% interlobular and intralobular space
4	Severe >50% interlobular and intralobular space
	**Severity of inflammation: inflammatory cell infiltration**
0	Absent
1	Minimal: rarely observed
2	Mild: 25–50% lobules in parenchyma
3	Moderate: 50–75% lobules in parenchyma
4	Severe: >75% lobules in parenchyma
	**Severity of acinar cell injury: vacuolization**
0	Absent
1	Minimal: rarely observed
2	Mild: <25% lobules displaying increase in vacuolization
3	Moderate: 25–50% lobules displaying increase in vacuolization
4	Severe: >50% lobules displaying increase in vacuolization
	**Severity of necrosis**
0	Absent
1	Minimal: rare
2	Mild: <5 necrotic cells
3	Moderate: 5–15 necrotic cells
4	Severe: >15 necrotic cells

**Table 2:** Histological scoring system for recovery phase in mice with cerulein-induced acute pancreatitis (2–7 days) after initial injection.

Score	Acinar Cell Repair (20× Objective Field)
0	Absent
1	<25% lobules
2	25–50% lobules
3	50–75% lobules
4	>75% lobules

## Data Availability

Data is contained within the article or Supplementary Material.

## Supplementary Materials

The following are available online at https://www.mdpi.com/2073-4409/10/4/728/s1, Figure S1: *Mfge8* knockout status does not affect pancreatic development in mice. Figure S2: Mfge8 KO mice exhibited delayed recovery of pancreatic injury in cerulein-induced pancreatitis.
