# Supplementary material for: MFG-E8 Plays an Important Role in Attenuating Cerulein-Induced Acute Pancreatitis in Mice
Source: Cells. 2021 Mar 25;10(4):728. doi: 10.3390/cells10040728 (PMC8064467; doi:10.3390/cells10040728)
Supplement: Supplementary file 1 [file cells-10-00728-s001.pdf]

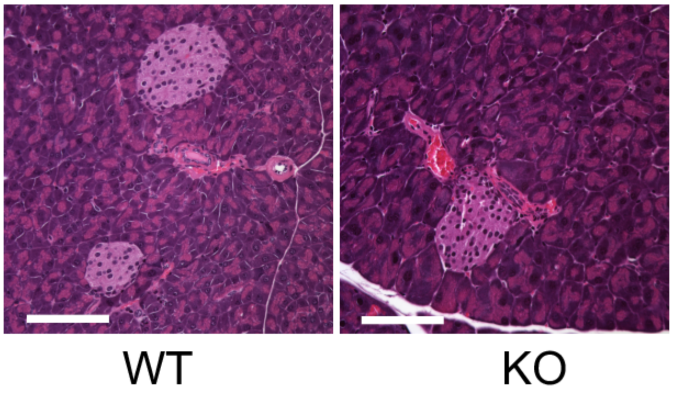

Supplementary Figure S1. *Mfge8* knockout status does not affect pancreatic development in mice. Pancreatic tissues from C57BL/6J WT and *Mfge8* KO mice (male, 8 – 10 weeks old) were processed for routine histology. Sections (5 μm) of pancreas tissues were processed for H&E staining and images are shown here. Scale bar: 100 μm.

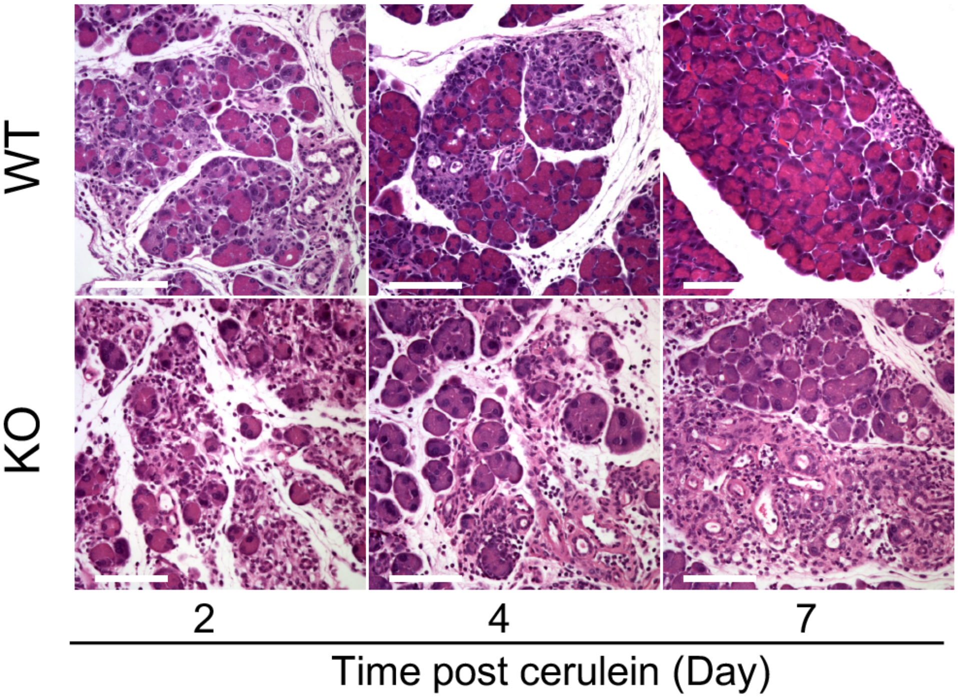

Supplementary Figure S2. *Mfge8 KO mice exhibited delayed recovery of pancreatic injury in cerulein-induced pancreatitis*. WT and *Mfge8* KO mice (male, 8 – 10 weeks old) were subjected to cerulein treatment as described in the Methods. Animals were euthanized at indicated timepoints. Representative histological images of pancreas (20X). H&E stain. Scale bar: 100 μm.
